# *Dirofilaria repens* in dogs and humans in Lithuania

**DOI:** 10.1186/s13071-019-3406-y

**Published:** 2019-04-18

**Authors:** Vytautas Sabūnas, Jana Radzijevskaja, Povilas Sakalauskas, Saulius Petkevičius, Birutė Karvelienė, Jolanta Žiliukienė, Indrė Lipatova, Algimantas Paulauskas

**Affiliations:** 1 Department of Biology, Faculty of Natural Sciences, Vytautas Magnus University, Vileikos str. 8, LT-44404 Kaunas, Lithuania; 2 Linas Veterinary Clinic, Debreceno str. 5, 94175 Klaipėda, Lithuania; 3 Department of Veterinary Pathobiology, Veterinary Academy, Lithuanian University of Health Sciences, Tilžės str. 18, 47181 Kaunas, Lithuania; 4 National Public Health Surveillance Laboratory, Žolyno str. 36, 10210 Vilnius, Lithuania

**Keywords:** Dirofilariosis, Autochthonous, *Dirofilaria repens*, Dogs, Humans, *Wolbachia*, Lithuania

## Abstract

**Background:**

In Lithuania, the first case of canine subcutaneous dirofilariosis was recorded in 2010. Since then, an increasing number of cases of canine dirofilariosis have been documented in different veterinary clinics throughout the country. Human dirofilariosis was diagnosed in Lithuania for the first time in September 2011. However, to the authors’ knowledge, there are no published data on the presence and prevalence of autochthonous dirofilariosis in dogs and humans in the country. The present study provides information about the predominant species and prevalence of *Dirofilaria* in dogs and describes the cases of human dirofilariosis in Lithuania. It also outlines PCR detection of the bacterial endosymbiont *Wolbachia* that contributes to the inflammatory features of filarioid infection.

**Results:**

A total of 2280 blood samples and six adult worms from pet and shelter dogs were collected in the central and eastern regions of Lithuania in 2013–2015. Based on their morphological appearance, morphometric measurements and molecular analysis, all the adult nematodes were identified as *Dirofilaria repens.* The diagnosis of microfilariae in blood samples was based on blood smear analysis and Knott’s test. The PCR and sequence analysis of the ribosomal DNA ITS2 region and *cox*1 gene confirmed the presence of *D. repens*. Overall, 61 (2.7%) of the 2280 blood samples were found to be positive for the presence of *D. repens.* The infection rate of *D. repens* was significantly higher in shelter dogs (19.0%; 19/100) than in pet dogs (1.9%; 42/2180) (*χ*^2^ = 100.039, *df* = 1, *P* < 0.0001). Forty-nine DNA samples of *D. repens*-infected dogs were tested for the presence of the bacterial endosymbiont *Wolbachia* and, of these, 40 samples (81.6%) were found to be positive. Three ocular and six subcutaneous cases of human dirofilariosis were diagnosed in Lithuania in the period 2011–2018.

**Conclusions:**

To the authors’ knowledge, this is the first report of autochthonous *D. repens* infection in dogs and humans in Lithuania. The present data demonstrate that *D. repens* is the main etiological agent of dirofilariosis in Lithuania. The DNA of the filarioid endosymbiotic bacterium *Wolbachia* was detected in the vast majority of dogs infected with *D. repens*.

## Background

Dirofilariosis is an emerging vector-borne parasitic zoonotic infection caused by nematodes of the genus *Dirofilaria.* The parasites are transmitted by a large variety of mosquito species belonging to the Culicidae family, including some species of the Lithuanian mosquito fauna, i.e. *Culex pipiens* (*s.l.*), *Anopheles maculipennis* (*s.l.*) and *Aedes vexans* [1–3]. Carnivores are the definitive hosts. The majority of cases in humans and animals are caused by two *Dirofilaria* species, *Dirofilaria repens* and *Dirofilaria immitis*, both of which can infect numerous mammalian species [4].

Among mammalian hosts, domesticated dogs (*Canis familiaris*) function as reservoirs and are the most important source for human transmission of both species [4]. Dirofilariosis caused by *D. repens* and *D. immitis* has been also reported in wild carnivores, e.g. wolves (*Canis lupus*), red foxes (*Vulpes vulpes*) and golden jackals (*Canis aureus*) [5–7].

The life-cycle of *D. repens* comprises five larval stages. Adult *D. repens* worms in dogs produce the first-stage larvae (microfilariae) and release them into the bloodstream. Only mature helminths of the genus *Dirofilaria* can produce microfilariae [4].

Canine subcutaneous dirofilariosis (*D. repens*) is often considered asymptomatic, although in some cases the parasites cause subcutaneous nodules, while circulating microfilariae cause dermatological signs such as pruritus, erythema, alopecia, diffused dermatitis and itching [8].

The traditional picture of human dirofilariosis caused by *D. repens* is associated with subcutaneous nodules and ocular locations. However, there have been some reports of human dirofilariosis in unusual sites. Parasites have been found in the lungs, scrotum, penis, spermatic cord, epididymis and female mammary glands [9, 10]. In most human cases of ocular dirofilariosis caused by *D. repens*, the parasites have been found in nodules or cysts in the eye or in periocular tissues [10]. Only a few cases of humans showing circulating microfilariae have been reported. Due to incomplete development, humans are not suitable hosts for disease transmission [11–13].

Many filarioid nematode species harbour intracellular bacteria of the genus *Wolbachia* (*Rickettsiaceae*) [14]. *Wolbachia* is found in all filarioid life stages and is essential for embryogenesis, normal development, fertility and the long-term survival of the adult worm [15–17]. *Wolbachia* is released into the blood and interacts with host tissues when adult worms and microfilariae die, stimulating inflammation and inducing immune responses [16, 18].

In Lithuania, the first case of canine dirofilariosis was recorded in 2010 in the small animal clinic of the Veterinary Academy in Kaunas in central Lithuania [19]. Since then, a growing number of canine dirofilariosis cases have been documented in different veterinary clinics throughout the country. Human dirofilariosis was diagnosed in Lithuania for the first time in September 2011. However, to the authors’ knowledge, no data have been published about the presence and prevalence of autochthonous *D. repens* infection in dogs and humans in Lithuania. Therefore, the aim of the present study was to determine the predominant species and prevalence rate of dirofilariosis in dogs and to describe the cases of human dirofilariosis in Lithuania. A further objective of this work was to perform molecular detection of the bacterial endosymbiont *Wolbachia*, which plays an important role in *D. repens* biology and contributes to the inflammatory pathology of the infection.

## Methods

### Sample collection

#### Samples collected from dogs

In co-operation with veterinary clinics and animal shelters in Kaunas (central Lithuania) and Vilnius (eastern Lithuania), a total of 2280 dogs (2180 pet dogs and 100 shelter dogs) were investigated for the presence of microfilariae in their blood in 2013–2015. None of the dogs examined had been imported from endemic countries or had ever travelled outside Lithuania. Dogs younger than 6 months were excluded due to the long life-cycle of *Dirofilaria*. The sex, age and body size of the shelter dogs were recorded.

The shelter and pet dog blood samples were taken from the cephalic vein, collected in EDTA-containing vacutainers and then stored at 4 °C or -20 °C until DNA isolation.

The microfilariae were detected based on blood smear microscopy and the modified Knott’s test. PCR and sequence analysis were applied in order to confirm diagnosis and molecularly characterise the *Dirofilaria* species.

In six dogs presented in small animal clinics, the adult worms were removed from skin nodules using a surgical technique. Adult nematodes were identified by morphological and molecular methods.

#### Samples collected from humans

Data on human dirofilariosis cases (patients’ travel history and other anamnestic data) were collected from the National Public Health Surveillance Laboratory (NVSPL), where each of the nine human dirofilariosis cases during the period 2011–2018 had been registered. Adult worms were removed from human patients during surgical procedures by ophthalmologists and surgeons in different areas of Lithuania. Helminths were subsequently sent to the NVSPL for further investigation. Species identification was undertaken using morphological and morphometric analysis. To confirm the diagnosis in the first case (Patient no. 1; Table 1), helminths were sent for investigation to the Swiss Institute of Parasitology of the University of Zurich (SCUP).

**Table 1:** A summary of information about human infections of *D. repens* in Lithuania in the period 2011–2018

Patient no.	Sex	Age	Locality	Travelling history	Year	Location in host
1	Female	76	Kaunas	None	2011	Ocular
2	Female	55	Vilnius	Turkey	2012	Subcutaneous (head)
3	Male	7	Vilnius	None	2012	Subcutaneous (abdomen)
4	Female	66	Vilnius	None	2013	Subcutaneous (head)
5	Female	66	Ukmergė	None	2013	Subcutaneous (chest)
6	Female	76	Kaunas	None	2014	Ocular
7	Female	51	Vilnius	None	2014	Ocular
8	Male	79	Utena	None	2015	Subcutaneous (penis)
9	Male	28	Klaipėda	Germany, Poland	2018	Subcutaneous (chest)

### Canine blood smear

After blood collection, a thin blood smear was immediately prepared. The slides were air-dried, fixed with methanol and stained with Giemsa stain. Slides were observed for microfilaria by light microscopy at 100× and 500× (oil immersion) magnification [20].

### Modified Knott’s test

Briefly, 1 ml of EDTA blood was added to 9 ml of 2% formalin, mixed by inversion and centrifuged at 3000× *g* for 5 min. The supernatant was discarded. The sediment was mixed with 35 μl of 0.1% methylene blue and 20 μl of this mixture was observed using a light microscope [21].

### Morphological identification of adult worms collected from dogs

The collected adult nematodes were measured and morphologically studied using light microscopy. The parasites were identified by an evaluation of the macroscopic and microscopic characteristics. About 1 cm of the nematode cephalic and caudal end was prepared with 50% glycerol for transparent slides. The middle parts of the nematodes were used for molecular identification. The length and the distance between the oral opening and vulva (for females) or the length of the left and right spicule (for males) of the nematodes were measured for morphological nematode identification [22].

### Molecular detection

Molecular analysis was performed to differentiate between and accurately identify the filarioid species. DNA was isolated from 200 µl aliquots of EDTA blood using a GeneJet Whole Blood Genomic DNA Purification Kit (Thermo Fisher Scientific, Vilnius, Lithuania) according to the manufacturer’s instructions. DNA from mature helminths was extracted using a QIAamp DNA Mini Kit (Qiagen, Hilden, Germany) according to the manufacturer’s instructions.

To identify filarioid species, conventional PCR and pan-filarial primers (DIDR-F1, DIDR-R1) were used that amplify fragments of different length of the internal transcribed spacer region 2 (ITS2) of the ribosomal DNA from six different filarioid species (*D. repens*, *D. immitis*, *Acanthocheilonema reconditum*, *Acanthocheilonema dracunculoides*, *Brugia pahangi* and *Brugia malayi*) [23]. The PCR was carried out as described by Rishniw et al. [23]. The identification was performed based on 484 bp fragments for *D. repens.* Blood samples positive for microfilaria and adult nematode samples were then verified with a *D. repens-*specific primer set (DR COI-F1/DR COI-R1) based on partial (209 bp) amplification of cytochrome oxidase subunit 1 (*cox*1) gene, as described by Rishniw et al. [23].

### *Wolbachia* endosymbiont

Samples positive for *Dirofilaria* spp. nematodes in pet and shelter dogs were further analysed by PCR (primers 16SwolbF/16SwolbR3) of the *16S* rRNA gene fragment of *Wolbachia* endosymbiont bacteria [24, 25] in 2017. The specific products obtained of 1018 bp were considered a positive result.

### Sequence analysis

PCR products of *D. repens* (ITS2 region and *cox*1 gene) and positive *Wolbachia* (*16S* rRNA) samples were extracted from gel using a GeneJet Gel Extraction Kit (Thermo Fisher Scientific) and sent off for sequencing (Macrogen, Amsterdam, Netherlands). The DNA sequences obtained were analysed using the Mega software package v.6.05, and compared with the GenBank database, searching for similar sequences using BLAST. Phylogenetic trees were constructed using the maximum-likelihood (ML) and neighbour-joining (NJ) methods.

Sequences obtained from PCR products of the partial ITS2 (*n* = 1) region and *cox*1 (*n* = 3) gene of *D. repens* (derived from the blood samples of dogs and adult nematodes) and the partial *16S* rRNA gene of *Wolbachia* (*n* = 2) were deposited in GenBank under accession numbers MH469230, MH469227-MH469229 and MK050782-MK050783, respectively.

### Statistical analysis

Pearson’s Chi-square analysis was performed with *D. repens* infection in shelter dogs (0, negative; 1, positive) against independent variables, including sex, age (0.5–3 years; > 3–6 years; and > 6 years), and body size (small, ≤ 10 kg; medium-sized, > 10–25 kg; and large, > 25 kg) groups.

Statistical analysis was carried out using the statistical software IBM SPSS Statistics software v.23 for Windows (SPSS Inc., Chicago, IL, USA); *P* > 0.05 was considered insignificant.

## Results

**Fig. 1:**
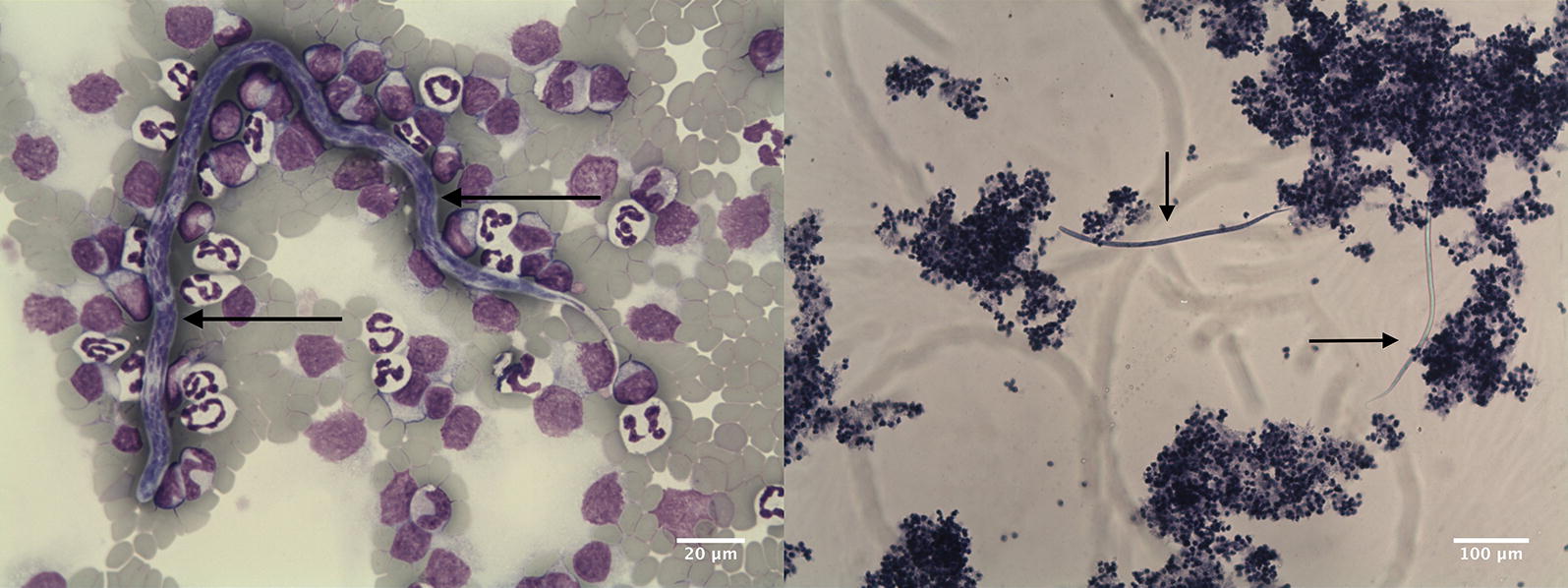
Microfilariae (arrows) of *D. repens* in **a** a blood smear at 500× magnification and **b** the modified Knott’s test at 100× magnification

Overall, 61 (2.7%, 95% CI: 2.3–3.9%) of the 2280 blood samples from pet and shelter dogs were found to be positive for the presence of microfilariae. A total of 42 (1.9%, 95% CI: 1.5–2.8%) of the 2180 pet dog blood smears (Fig. 1a) were positive for microfilariae. Using the modified Knott’s method (Fig. 1b), microfilariae were found in 19 (19%, 95% CI: 11.8–27.4%) of the 100 shelter dogs. The infection rate of *D. repens* parasites was significantly higher in shelter dogs (19.0%) than in pet dogs (1.9%) (*χ*^2^ = 100.039, *df* = 1, *P* < 0.0001).

The shelter dogs were 0.5 to 18 years-old, with a median age of 5 years (IQR 2.0–10.0). Infected animals were identified in all age groups: 0.5–3 years, prevalence of 18.4% (7/38); > 3–6 years, prevalence of 9.1% (2/22); and > 6 years, prevalence of 25.0% (10/40). The infection rate in males was 22.2% (10/45) and 16.4% (9/55) in females. There were no significant differences in prevalence (*P* > 0.05) between shelter dogs of different sexes and ages. Additionally, dogs in the medium (20.8%; 15/72) and large size (33.3%, 2/6) dog groups were more frequently infected than small dogs (3.7%, 1/22); however, body size was not a statistically significant factor (*χ*^2^ = 3.165, *df* = 1, *P* = 0.106; *χ*^2^ = 4.084, *df* = 1, *P* = 0.107, respectively).

**Fig. 2:**
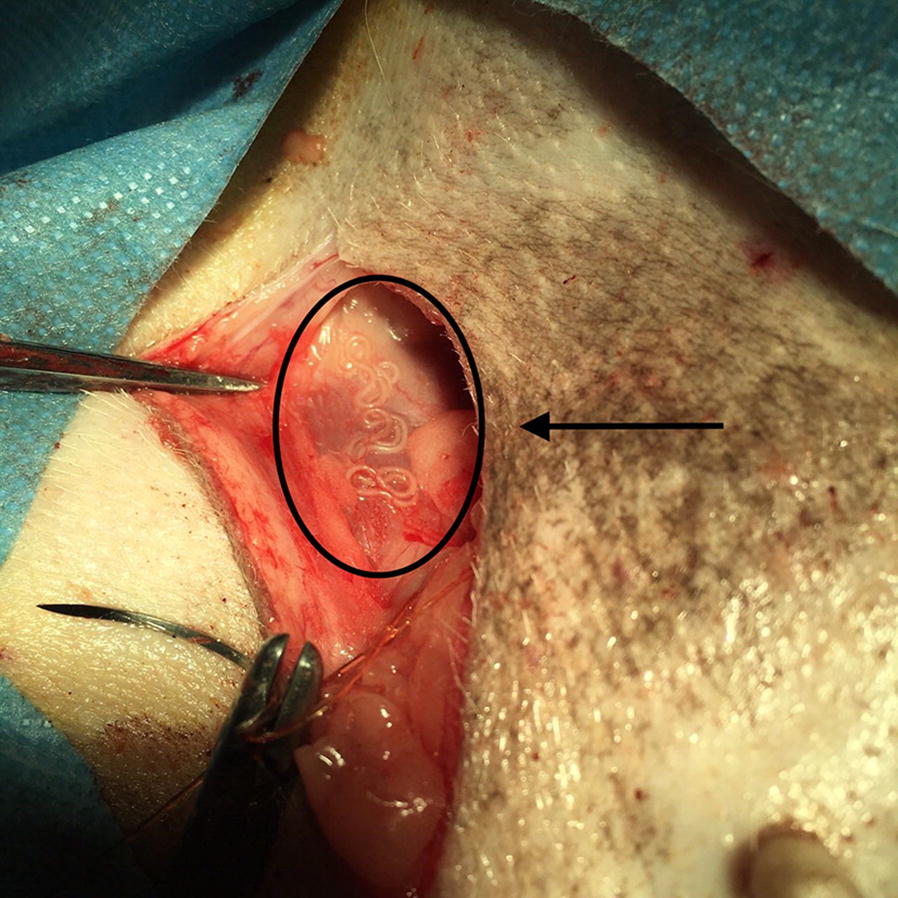
Subcutaneous localisation of an adult *D. repens* worm (arrow) in a French bulldog during neutering surgery

All measurements of adult worms removed from dogs (Fig. 2) were within the known range for *D. repens.* One male and five females were identified.

### Human cases

**Fig. 3:**
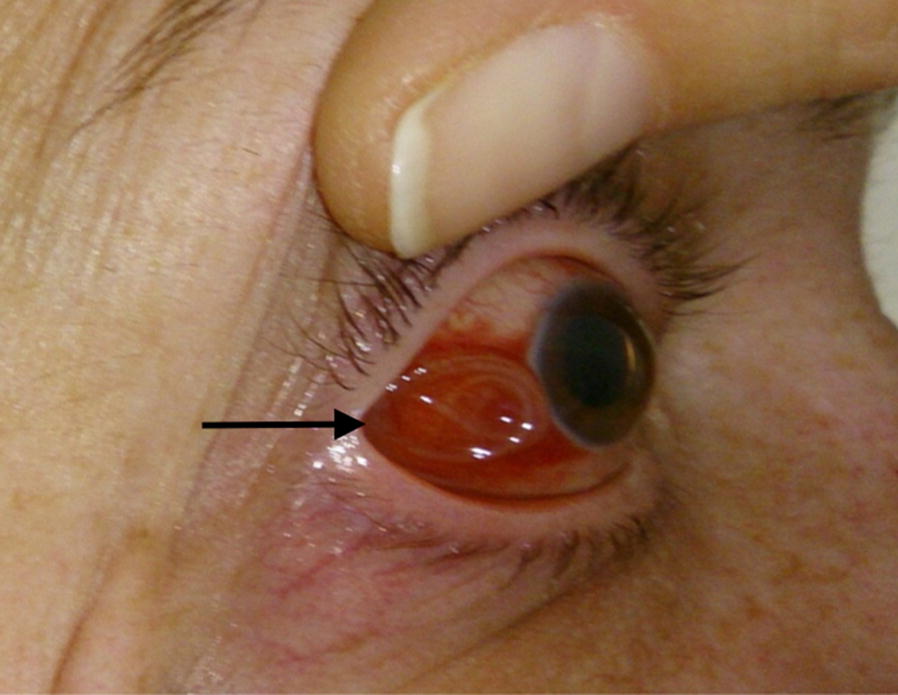
Subconjuctival localisation of a *D. repens* adult worm (arrow) in the human eye, patient no. 7 (Table 1)

Overall, nine cases of human dirofilariosis infection were observed between 2011 and 2018 in Lithuania (Table 1). In three cases worms were found in the ocular localisation, and in six cases in subcutaneous tissue (Fig. 3). Seven of the nine infected humans had not travelled outside Lithuania. However, two patients had travelled to endemic countries within the two years prior to diagnosis. All nine nematodes were identified as *D. repens.*

### Molecular and phylogenetic analysis

PCR amplification of the partial ITS2 region and *cox*1 gene confirmed the *D. repens* species in all the positive samples (62 blood and 6 adult worm samples). No other filarioid species were found during this study.

**Fig. 4:**
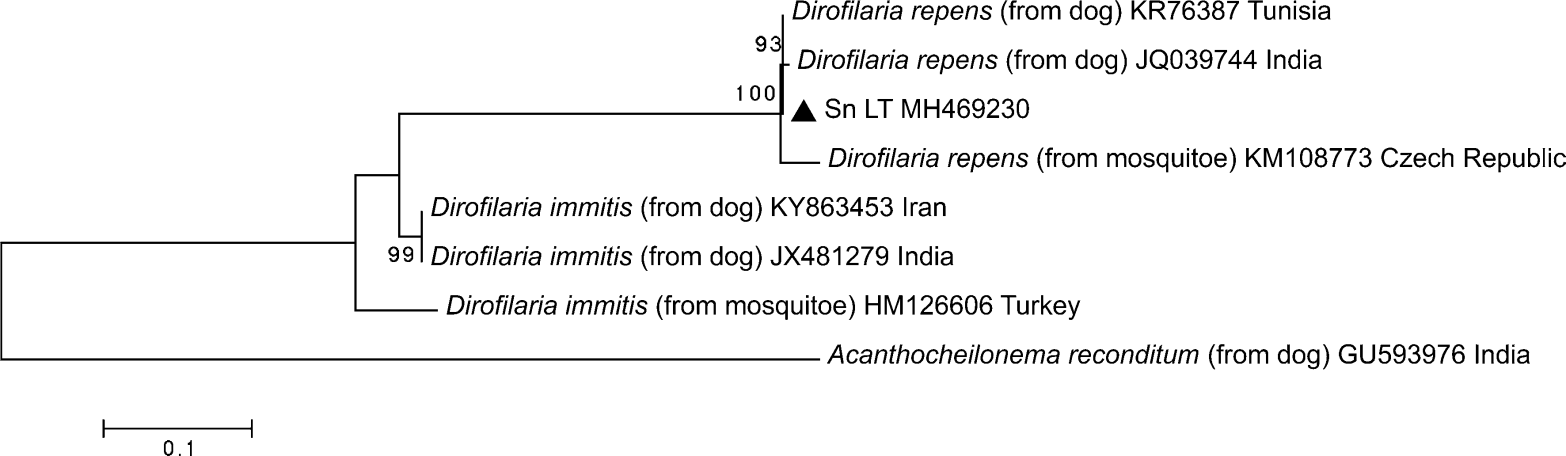
Phylogenetic tree of filarioid nematodes based on ITS2 rDNA sequences created using the maximum-likelihood (ML) method and bootstrap analysis of 1000 replicates. The representative sequences obtained in this study are marked with a black triangle

Ten randomly selected PCR products of *D. repens* for the ITS2 region and *cox*1 gene were purified and sequenced to confirm the PCR results. The sequence analysis of the partial ITS2 region showed that Lithuanian *D. repens* isolates derived from the blood of dogs were 100% identical to each other and to the corresponding *D. repens* sequence from Tunisia deposited in GenBank (KR676387) and shared 99% similarity (with one to three nucleotides difference) with *D. repens* isolates from the Czech Republic and India (Fig. 4).

**Fig. 5:**
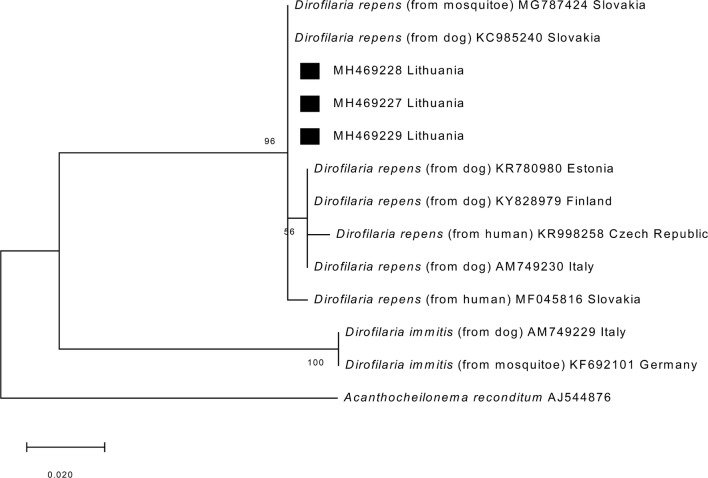
A phylogenetic tree of filarioid nematodes based on *cox*1 gene sequences created using the maximum likelihood method and bootstrap analysis of 1000 replicates. The representative sequences obtained in this study are marked with a black square (MH469227: representative of sequences obtained from canine blood; MH469228, MH469229: sequences obtained from two adult nematodes)

**Fig. 6:**
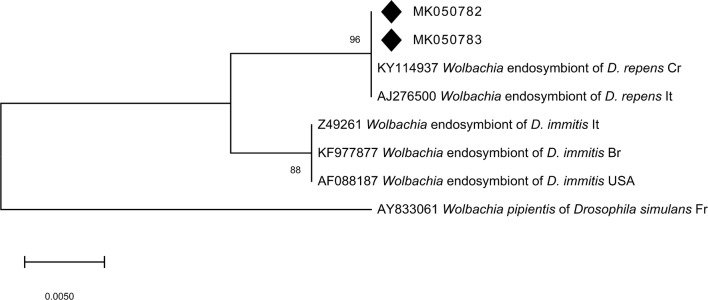
Neighbour-joining phylogenetic tree for the partial *16S* rRNA gene of the *Wolbachia* endosymbiont. The phylogenetic tree was created using the Kimura 2-parameter model with a bootstrap analysis of 1000 replicates. Sequences with accession numbers were taken from GenBank for comparison. The identification source (host) and country codes are provided. Samples sequenced in the present study are marked with a black rhombus

The phylogenetic analysis of the partial *cox*1 gene for *D. repens* isolates from mosquitoes, dogs and humans revealed four different *cox*1 variants. Analysed sequences included three variable sites. Lithuanian *D. repens cox*1 sequences derived from canine blood and adult nematode samples were identical to each other and showed 99–100% similarity to other European strains of *D. repens* (GenBank: MG787424, KC985240, KR780980, KY828979, KR998258, AM749230 and MF045816) (Fig. 5). *Wolbachia* endobacteria were found to be positive for *D. repens* in 40 of the 49 samples (81.6%, 95% CI: 69.4–91.3%). Sequence analysis of the partial *16S* rRNA gene showed that the obtained sequences were identical, displaying homology (100%) with sequences of *Wolbachia* endosymbiont of *D. repens* in Croatia (accession number KY114937) and Italy (accession number AJ276500). The phylogenetic analyses of *16S* rRNA sequences confirmed that *Wolbachia* endosymbionts of *Dirofilaria* separated according to the host species (Fig. 6).

## Discussion

Until 2001, canine *D. repens* infection had only been reported in southern European countries [26]. In recent years, however, autochthonous cases of dirofilariosis in dogs caused by *D. repens* have been reported in previously non-endemic countries with the following prevalences: 13.0–49.2% in Serbia [27–30]; 11.7–37.5% in Poland [31–34]; 20.0–34.5% in Slovakia [35–37]; 4.3–20.5% in Romania [38–41]; 15.8% in Latvia [42]; 14.0% in Hungary [43]; 9.0% in the Czech Republic [44]; and 6.8% in Germany [45]. Autochthonous *D. repens* cases have also been recorded in the Netherlands [46], Austria [47, 48], Belarus [49] and Ukraine [50]. New *D. repens* infections in northernmost Europe, which are likely to be autochthonous, have been reported in Estonia [51] and Finland [52]. Climatic changes, global warming and the movement of dogs across Europe are the main factors influencing the continuing spread of *Dirofilaria* in European countries [11, 53]. The geographical distribution of dirofilariosis depends on the presence of the definitive host and appropriate vectors in the area. Environmental temperature is one of the most important abiotic factors influencing the survival of insects and allowing the development of *Dirofilaria* into the infective stage in the mosquito. Various species of the genera *Aedes*, *Ochlerotatus*, *Culex*, *Culiseta* and *Anopheles* are potential vectors of *D. repens* [16, 54] in Europe. Currently, 37 mosquito species are known to be present in Lithuania [1, 55]. Of these, *Aedes vexans*, *Culiseta annulata*, *Culex pipiens*, *Anopheles maculipennis*, *Ochlerotatus caspius* and *Ochlerotatus excrucians* are potential vectors for *D. repens* in Lithuania [16, 56–58]. To the best of the authors’ knowledge, there is no information about the prevalence of *D. repens* in Lithuanian mosquitoes, therefore further investigation is needed for accurate determination of competent vectors for *D. repens* in Lithuania. Environmental and climatic changes are now strongly influencing the activity patterns of mosquitoes in temperate areas, allowing more generations of vectors each year [54]. Furthermore, climate change and global warming are factors driving the disease vectors’ ability to invade new areas in which they could potentially transmit pathogens.

This study reports the prevalence and molecular characterisation of *D. repens* in dogs in the Baltic region (Lithuania). It is worth mentioning that the examined dogs had never travelled outside Lithuania. The overall prevalence of *D. repens* infection in pet and shelter dogs was 2.7%, with a significantly higher prevalence detected in shelter dogs (19.0%). A similar prevalence of infection among shelter dogs has been obtained in the neighbouring countries of Latvia (15.8%; 22/139) [42] and Poland (11.3%; 14/124) [59], while a lower incidence of *D. repens* infection has been detected in Italy (3.4%; 4/118) [60] and Romania (2.2%; 2/92) [39]. Several factors could explain the difference in the prevalence of infection in shelter and pet dogs detected in the present study. The detection method may affect the sensitivity of the parasite detection. Microfilariae in blood samples from the pet dogs were detected based on blood smear analysis, while in shelter dogs, Knott’s test was applied for parasite detection. Knott’s test is a more sensitive test because it concentrates the microfilaria, making it less likely to be missed during microscopic examination. Perhaps the most important factor is that shelter dogs are most often caught roaming free in streets and are in greater contact with vectors than dogs living in the human environment. Furthermore, shelter dogs do not usually receive prophylactic treatments and are therefore at a higher risk of infection. These findings suggest that shelter dogs may serve as major reservoirs for *D. repens* in urban areas in Lithuania.

Given that dogs are the most important source for human transmission of dirofilariosis [4], there should be stricter rules around the use of preventive measures and regulations for free-ranging dogs.

Human dirofilariosis has been diagnosed in five different areas in eastern, central, western and north-eastern Lithuania: Vilnius, Kaunas, Klaipėda, Ukmergė and Utena. Five human cases of autochthonous *D. repens* have been found in the central and eastern parts of the country (Kaunas, Vilnius) in areas where dirofilariosis has been diagnosed in dogs. A total of seven out of the nine people investigated had never travelled to endemic countries. These data show that human subcutaneous dirofilariosis is also endemic in Lithuania. Human *Dirofilaria* infections are sporadic in Lithuania and in neighbouring countries, where the first cases of dirofilariosis were noted at a similar time [61–63]. In the past few decades, the number of human infections in previously endemic countries has increased dramatically [9, 16, 50, 64]. The current epidemiological situation of dirofilariosis in Europe, including in the Baltic countries, suggests that previously non-endemic countries should expect an increase in human infections in future.

Both filarioid species (*D. immitis* and *D. repens*) require the same temperature and the same time interval for incubation in the same vector species under laboratory conditions [64, 65]. Recently reported autochthonous cases of heartworm infection in Central European countries [32, 44, 66, 67] and the documented imported case in the Baltic countries [68] demonstrate that veterinary practitioners should expect the expansion of life-threatening *D. immitis* in north-eastern Europe.

Arthropod-borne diseases are increasing in Lithuania [69, 70] and other European countries [71]. The samples examined in this study were also tested for the presence of tick-borne pathogens such as *Anaplasma phagocytophilum* and *Babesia canis* and possible co-infection with *D. repens* (data not shown). Out of 2180 blood samples from pet dogs, 3 (0.1%) were positive for *A. phagocytophilum* and 38 (1.7%) were positive for *B. canis.* Of 42 *D. repens*-positive samples, nine contained co-infection with *B. canis* and in one sample a triple-infection with *D. repens*, *B. canis* and *A. phagocytophilum* was detected. These findings suggest that co-infections with anaplasmosis and babesiosis in patients infected with *Dirofilaria* are suspected. Co-infected cases are complicated for practitioners, and may cause failures in diagnosis, treatment and prognosis [72–74].

More studies on *Wolbachia* have been carried out around the world on arthropods than on filarioids (family Onchocercidae) [17, 18, 75]. Based on the data thus far obtained, endosymbiontic *Wolbachia* has been detected in at least 28 filarioid species [17, 76]. To the authors’ knowledge, this is the first report on the detection of endosymbiontic *Wolbachia* bacteria in Lithuanian dogs infected with *D. repens*. *Wolbachia* DNA was detected in the blood of more than 80.0% of all the dogs infected with *Dirofilaria*. According to previous reports, *Wolbachia* endosymbionts are detected in 30.6–52.6% of dogs infected with *D. repens* or *D. immitis* in Europe [24, 77]. Meanwhile in the Asian part of Turkey, *Wolbachia* has been detected in just 6.2% of the tested dogs infected with *D. immitis* [78]. Phylogenetic analyses indicate that *Wolbachia* detected in *D. repens* and *D. immitis* represents one group [15, 73, 74, 79]. These studies will drive further investigations to improve understanding of the importance of symbiosis between *Wolbachia* and *Dirofilaria*. *Wolbachia* could therefore be used as a target of antibiotic therapy [16, 25, 79].

## Conclusions

To the authors’ knowledge, this is the first report of autochthonous *D. repens* infection in dogs and humans in Lithuania. The present data extend the knowledge about the distribution of *D. repens* in shelter dogs and dogs living in the human environment and demonstrated that *D. repens* is the main etiological agent of dirofilariosis in Lithuania. The DNA of the filarioid endosymbiotic bacterium *Wolbachia* was detected in the vast majority of dogs infected with *D. repens*.

## Abbreviations

EDTA: ethylenediamine tetraacetic acid
ITS: internal transcribed spacer
PCR: polymerase chain reaction
*16S* rDNA: *16S* ribosomal RNA gene
*cox*1: cytochrome *c* oxidase subunit 1
